# P-368. Population Pharmacokinetic Analysis of Islatravir and the Impact of Intrinsic Factors in Participants with HIV-1

**DOI:** 10.1093/ofid/ofaf695.586

**Published:** 2026-01-11

**Authors:** Michelle M Pham, Irene Bae, Michelle C Fox, Lihong Du, Seth H Robey, Munjal Patel, Martin Johnson, Bill Poland, Stephanie O Klopfer, Ryan C Vargo, Brian M Maas

**Affiliations:** Merck & Co., Inc; Certara, Princeton, New Jersey; Merck & Co., Inc; Merck & Co., Inc; Merck & Co., Inc; Merck & Co., Inc; Allucent, Bracknell, England, United Kingdom; Certara, Princeton, New Jersey; Merck & Co., Inc; Merck & Co., Inc; Merck & Co., Inc

## Abstract

**Background:**

Islatravir (ISL) is an investigational nucleoside reverse transcriptase translocation inhibitor being evaluated in combination regimens for the treatment of HIV-1. A population pharmacokinetic (PopPK) model was constructed to describe the pharmacokinetics (PK) of plasma ISL and its intracellular active anabolite ISL-triphosphate (ISL-TP). This work was aimed to provide dosing recommendations for ISL for the treatment of HIV-1 and identify the ISL-TP exposure associated with activity against wild-type virus and M184I/V variants.

**Figure:**
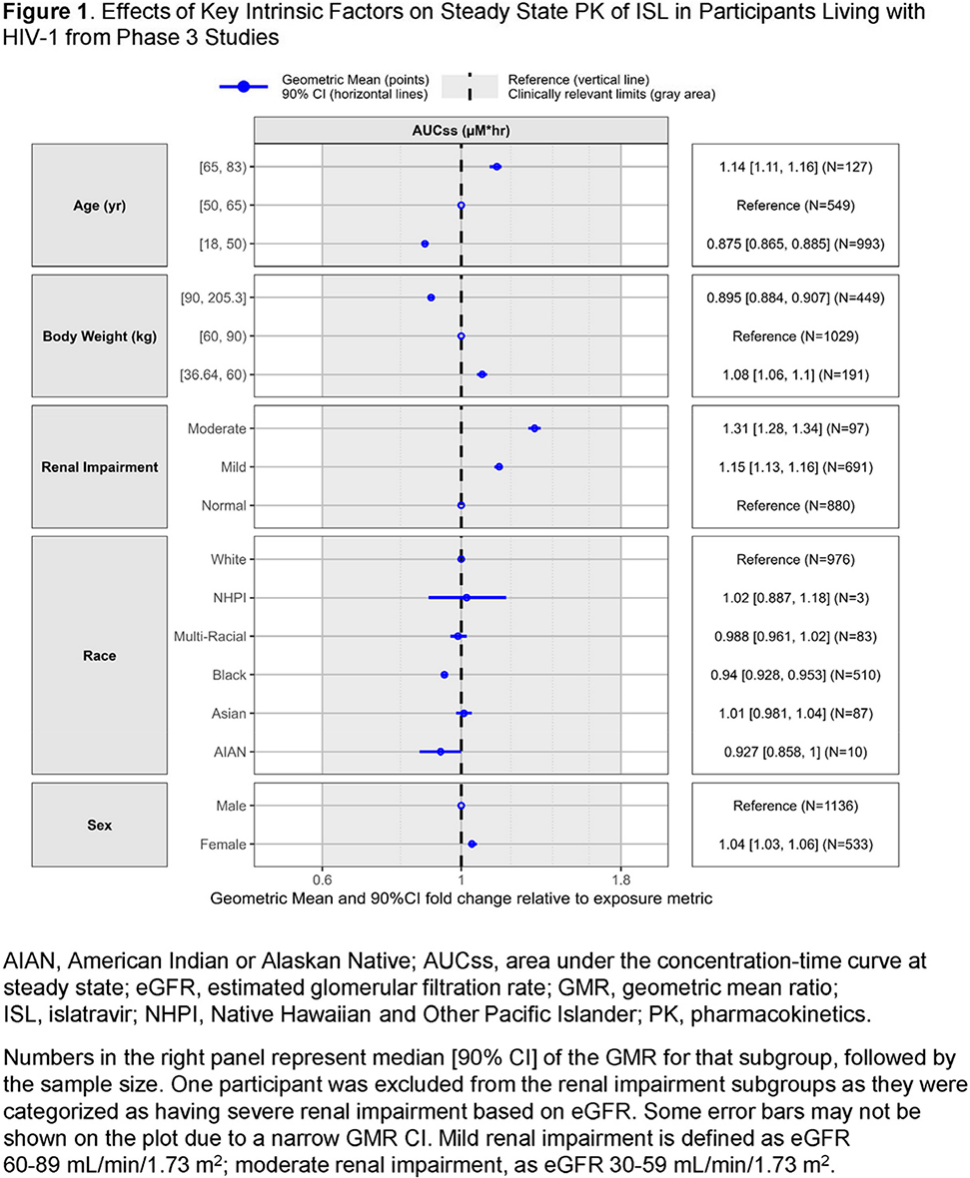

**Figure:**
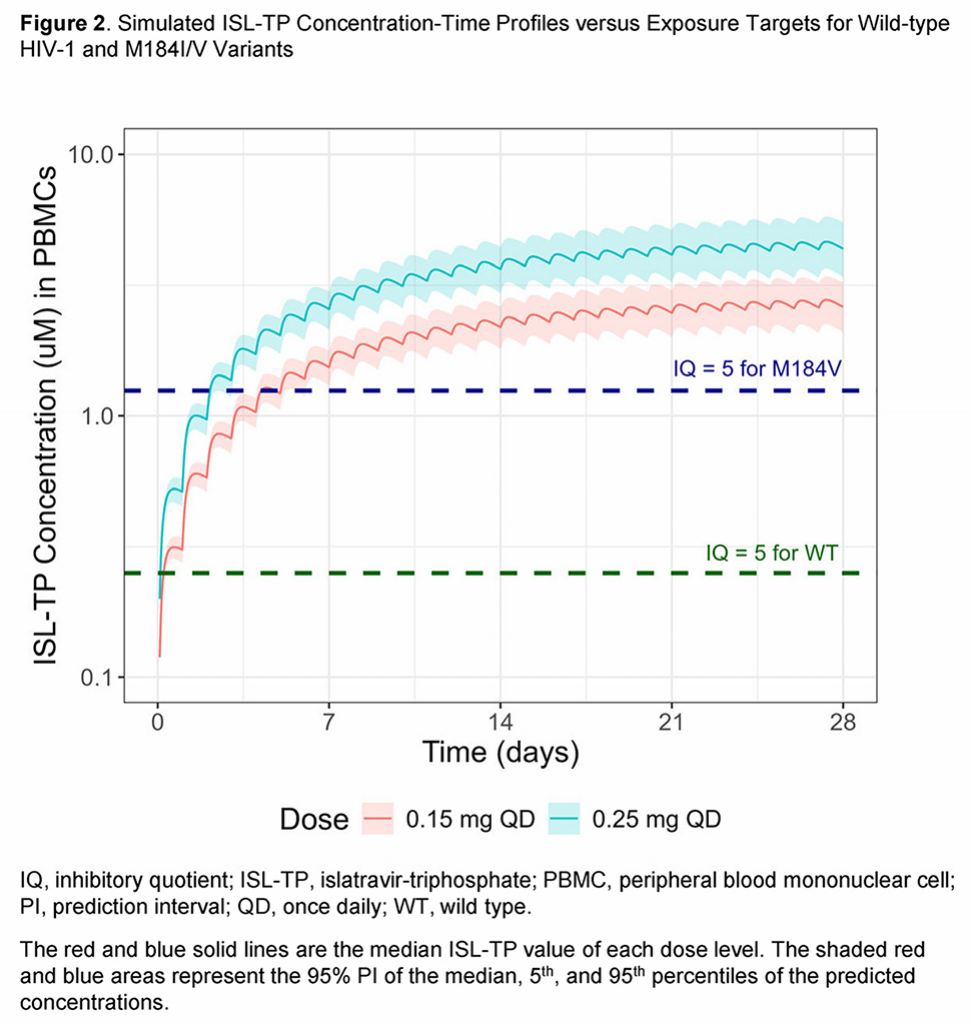

**Methods:**

Plasma and peripheral blood mononuclear cell (PBMC) samples were analyzed from adult and pediatric participants with and without HIV-1 receiving ISL across 18 studies, including two Phase 3 studies being conducted to evaluate oral ISL 0.25 mg once daily (QD) in combination with doravirine 100 mg. A PopPK model was developed and used to 1) evaluate the clinical relevance of intrinsic factors for ISL PK (ie, age, weight, sex at birth, race/ethnicity, mild/moderate renal impairment) and 2) determine the lower clinical bound based on inhibitory quotient (IQ) targets (defined as the trough ISL-TP concentration / 5 × the *in vitro* ISL ISL-TP EC_50_).

**Results:**

The analysis included 16,230 plasma ISL and 3999 PBMC ISL-TP concentrations from 2321 adult participants. Plasma ISL and PBMC ISL-TP were best characterized using a 5-compartment model with linear absorption and elimination. Age, weight, estimated glomerular filtration rate and disease status (without/with HIV) were statistically significant covariates on the PK of ISL; however, none of these intrinsic factors correlated with clinically significant changes in ISL exposure (Figure 1). Final model-based simulations showed that ISL 0.15 mg QD (corresponding to a 0.6-fold reduction in ISL AUC_0-24_, compared with ISL 0.25 mg) results in an ISL-TP C_24_ that meets IQ targets for wild-type virus and M184I/V variants (Figure 2).

**Conclusion:**

The PopPK model captured the PK of ISL and ISL-TP well. ISL can be administered to virologically suppressed adults without regard to age, weight, sex, race/ethnicity, or mild/moderate renal impairment. No reduction in efficacy is expected at ISL exposures at a geometric mean ratio of 0.6-fold of those produced by the proposed clinical dose of 0.25 mg QD.

**Disclosures:**

Michelle M. Pham, PhD, Merck & Co., Inc: Full Time employee Michelle C. Fox, MD, Merck & Co., Inc.: Employment|Merck & Co., Inc.: Stocks/Bonds (Public Company) Lihong Du, PhD, Merck & Co., Inc.: Employment|Merck & Co., Inc.: Stocks/Bonds (Public Company) Seth H. Robey, PhD, Merck & Co.,Inc.: Employment|Merck & Co.,Inc.: Stocks/Bonds (Public Company) Munjal Patel, PhD, Merck & Co., Inc.: Employment|Merck & Co., Inc.: Stocks/Bonds (Public Company) Martin Johnson, PhD, Allucent: Stocks/Bonds (Public Company) Stephanie O. Klopfer, PhD, Merck & Co., Inc: Employment|Merck & Co., Inc: Stocks/Bonds (Public Company) Ryan C. Vargo, PhD, Merck and Co.: Full time employee|Merck and Co.: Stocks/Bonds (Public Company) Brian M. Maas, PharmD, Merck & Co. Inc.: Employment|Merck & Co. Inc.: Stocks/Bonds (Public Company)

